# Context-dependent compensation among phosphatidylserine-recognition receptors

**DOI:** 10.1038/s41598-017-15191-1

**Published:** 2017-11-07

**Authors:** Kristen K. Penberthy, Claudia Rival, Laura S. Shankman, Michael H. Raymond, Jianye Zhang, Justin S. A. Perry, Chang Sup Lee, Claudia Z. Han, Suna Onengut-Gumuscu, Krzysztof Palczewski, Jeffrey J. Lysiak, Kodi S. Ravichandran

**Affiliations:** 1 Center for Cell Clearance, University of Virginia, Charlottesville, VA USA; 2 Department of Microbiology, Immunology and Cancer Biology, University of Virginia, Charlottesville, VA USA; 3 Department of Urology, University of Virginia, Charlottesville, VA USA; 4 Department of Neuroscience, University of Virginia, Charlottesville, VA USA; 5 Department of Pharmacology and Cleveland Center for Membrane and Structural Biology, School of Medicine, Case Western Reserve University, Cleveland, OH USA; 6 Center for Public Health Genomics, University of Virginia, Charlottesville, VA USA; 7 Inflammation Research Center, VIB, and the Department of Biomedical molecular Biology, Ghent University, Ghent, Belgium; 8 College of Pharmacy and Research Institute of Pharmaceutical Sciences, Gyeongsang National University, 501 Jinju-daero, Jinju, Gyeongnam, 52828 Korea

## Abstract

Phagocytes express multiple phosphatidylserine (PtdSer) receptors that recognize apoptotic cells. It is unknown whether these receptors are interchangeable or if they play unique roles during cell clearance. Loss of the PtdSer receptor *Mertk* is associated with apoptotic corpse accumulation in the testes and degeneration of photoreceptors in the eye. Both phenotypes are linked to impaired phagocytosis by specialized phagocytes: Sertoli cells and the retinal pigmented epithelium (RPE). Here, we overexpressed the PtdSer receptor BAI1 in mice lacking MerTK (*Mertk* ^−/−^ *Bai1* ^*Tg*^) to evaluate PtdSer receptor compensation *in vivo*. While *Bai1* overexpression rescues clearance of apoptotic germ cells in the testes of *Mertk* ^−/−^ mice it fails to enhance RPE phagocytosis or prevent photoreceptor degeneration. To determine why MerTK is critical to RPE function, we examined visual cycle intermediates and performed unbiased RNAseq analysis of RPE from *Mertk* ^+/+^ and *Mertk* ^−/−^ mice. Prior to the onset of photoreceptor degeneration, *Mertk* ^−/−^ mice had less accumulation of retinyl esters and dysregulation of a striking array of genes, including genes related to phagocytosis, metabolism, and retinal disease in humans. Collectively, these experiments establish that not all phagocytic receptors are functionally equal, and that compensation among specific engulfment receptors is context and tissue dependent.

## Introduction

Cell death is a crucial part of life. Each day, billions of cells in the human body undergo apoptotic cell death and must be cleared^1–3^. In many tissues, dying cells are cleared by phagocytes that engulf and digest the apoptotic corpse^3–5^. Impediments to apoptotic cell clearance can lead to chronic inflammation and autoimmunity^6–9^. Apoptotic cell clearance is stimulated upon apoptotic cell exposure of the ‘eat-me’ signal phosphatidylserine (PtdSer) and its subsequent recognition by PtdSer receptors on phagocytes^10–12^. Phagocytes express a multitude of PtdSer receptors, yet, despite extensive research on apoptotic cell recognition and clearance, the relative importance of PtdSer receptors in a given phagocyte or tissue context is unclear^4,13^.

PtdSer receptors include many different families of proteins including integrins, the BAI family of GPCRs, the TIM family, and the TAM family of receptor tyrosine kinases^2,3,14–20^. These protein families are structurally diverse and are related only by their shared ligand, PtdSer^3–5^. Given the importance of apoptotic cell clearance to health and homeostasis, it is not surprising that many different receptors have evolved. However, the loss of a single receptor often causes deficits in apoptotic cell clearance, suggesting that the presence of many receptors cannot be explained as a simple redundancy mechanism^1,7,9,10,17,18^. Thus, the question of whether PtdSer receptors are interchangeable or unique in cell clearance remains unclear. To address this question, we considered designing a mouse model in which one PtdSer receptor is overexpressed in the absence of another in order to assess functional compensation *in vivo*.

In most tissues, evaluation of phagocytosis *in vivo* is complicated by the unpredictable timing of apoptosis and phagocytic events. Two exceptions to this rule are tissues where phagocytosis is mediated by ‘specialized’ phagocytes: the testes and the retina^5,14,18^. Specialized phagocytes are epithelial-derived, mitotically-quiescent cells^21–23^. Currently, two best known examples of specialized phagocytes are the retinal pigmented epithelium (RPE) of the eye and the Sertoli cells of the testes^5^. Sertoli cell phagocytosis is easily studied in the testes as apoptosis of developing germ cells occurs with sufficient regularity to quantify apoptotic corpse accumulation. Similarly, RPE phagocytosis is conducive to *in vivo* analysis as RPE phagocytosis is circadian-regulated and occurs daily around the time of light onset^14,21,24,25^. Furthermore, loss of a single PtdSer receptor, MerTK, leads to phagocytic defects in both Sertoli cells and the RPE^10,14,21,26^. *Mertk* ^−/−^ mice exhibit apoptotic corpse accumulation in the testes and profound retinal degeneration^10,26,27^. Thus, *Mertk* ^−/−^ mice are uniquely suited for *in vivo* evaluation of compensation among PtdSer receptors. Indeed, an elegant study by Vollrath et. al. determined that enhanced of expression of *Tyro3* can rescue the defective RPE phagocytosis in *Mertk* ^−/−^ mice^27^. Therefore, we asked whether a PtdSer receptor from a completely different family would be capable of the same rescue. To answer this question, we overexpressed the PtdSer receptor BAI1 in *Mertk* ^−/−^ mice (*Mertk* ^−/−^ *Bai1* ^*Tg*^).

Specialized phagocytes perform a multitude of supportive functions within the tissue. Though the testes and retina are highly disparate at first glance, Sertoli cells and RPE share several key features, including: epithelial derivation, mitotic quiescence, formation of a blood-tissue barrier, maintenance of immunological privilege, and as stated above, phagocytosis^22,23,26^. As, phagocytes, Sertoli cells are responsible for clearing germ cells that undergo apoptosis during spermatogenesis and the residual-body of cytoplasm that is removed from maturing sperm during spermiation^25,28^. RPE have a slightly different phagocytic function, unlike Sertoli cells which mediate corpse clearance, the RPE ‘trim’ the adjacent photoreceptors in a PtdSer dependent manner^13,21,26,29^. This RPE-mediated pruning of the photoreceptors occurs daily and is a waste-removal mechanism, removing the photo-oxidative byproducts that accumulate during phototransduction^14,29,30^. *Mertk* ^−/−^ mice are born with a full complement of photoreceptors but exhibit early-onset photoreceptor degeneration due to impaired phagocytosis (pruning) of photoreceptor outer segments (POS)^1,14,21^.

Here, we observe that while the BAI1 transgene has the ability to reduce apoptotic corpse accumulation in the testes of *Mertk* ^−/−^ mice following testicular torsion, the phagocytic defect *in Mertk* ^−/−^ RPE is not compensated by transgenic overexpression of BAI1. When we evaluated the visual cycle (a function unique to RPE), we observed that MerTK expression impacted the visual cycle prior to the onset of retinal degeneration. In addition, RNAseq revealed that the expression of many genes, including those related to phagocytosis and metabolism and other forms of retinal disease, were dysregulated in *Mertk* ^−/−^ RPE prior to the onset of retinal degeneration. Collectively, these findings suggest that PtdSer receptor functionality is contingent on tissue and context, and that while they can compensate for each other in certain contexts, they also have unique roles where they are not interchangeable.

## Results

### BAI1-transgene reduces corpse accumulation in the testes of Mertk^−/−^ mice

To determine whether distinct PtdSer receptors play unique roles in the process of engulfment, we designed a genetic approach to determine whether the overexpression of one receptor could rescue for the loss of another. We crossed MerTK null (*Mertk* ^−/−^) and BAI1-overexpressing (*Bai1* ^*Tg*^) mice to generate *Mertk* ^−/−^ *Bai1* ^*Tg*^ mice. The rationale for choosing *Mertk* ^−/−^ mice is that they have two *in vivo* phenotypes associated with impaired phagocytosis. First, *Mertk* ^−/−^ mice exhibit accumulation of apoptotic germ cells in the testes due to impaired Sertoli cell phagocytosis^10,31^. Second, these mice exhibit profound retinal degeneration due to failed clearance of photoreceptor outer segments (POS) by the RPE^2,14,32^. We elected to overexpress *Bai1* in an attempt to rescue the phenotypes in *Mertk* ^−/−^ mice, as BAI1 overexpression can enhance PtdSer-dependent apoptotic cell clearance by multiple phagocytes, including intestinal epithelial cells and Sertoli cells. Furthermore, *Bai1* ^*Tg*^ mice had been previously generated and characterized^1,5,18,33^.

Sertoli cells are the specialized phagocytes of the testes and promote routine phagocytosis of apoptotic germ cells. Sertoli cells utilize both BAI1 and MerTK during the phagocytosis of apoptotic germ cells^1,10^. *Mertk* ^−/−^ mice exhibit accumulation of apoptotic corpses at baseline^10^ and *Bai1* ^−/−^ mice exhibit apoptotic corpse accumulation following testicular torsion^1^. Furthermore, *Bai1* ^*Tg*^ mice exhibit a decrease in corpse accumulation following torsion^1^. Given the endogenous role for MerTK and BAI1 in the testes, we initially tested PtdSer receptor compensation in *Mertk* ^−/−^ *Bai1* ^*Tg*^ mice in the context of Sertoli cells.

**Figure 1:**
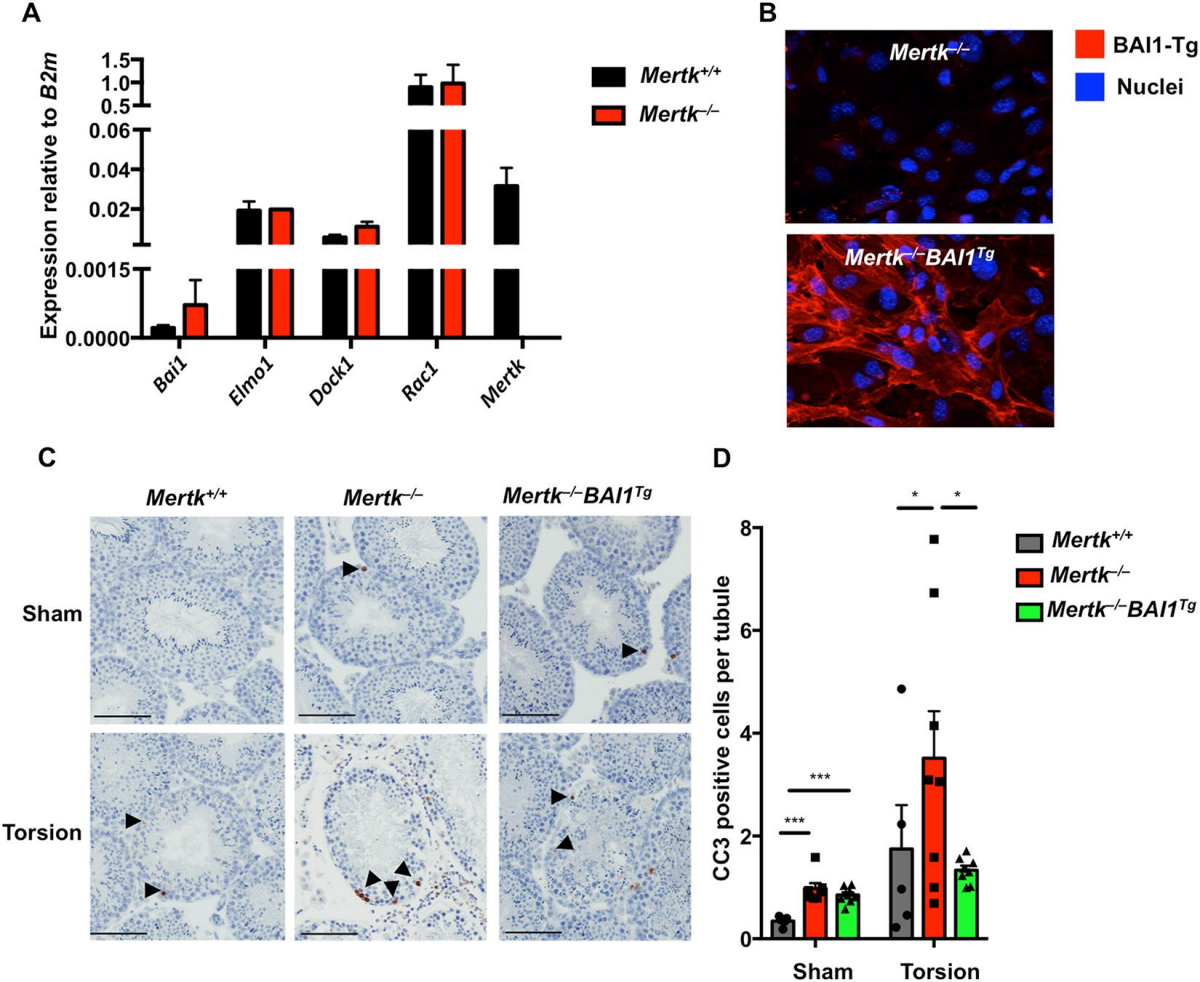
*Bai1* ^*Tg*^ reduces accumulation of apoptotic corpses in *Mertk* ^−/−^ mice post-torsion. (**A**) Sertoli cell expression of *Bai1*, BAI1 signaling pathway genes, and *Mertk* were analyzed by quantitative RT-PCR. Sertoli cells were isolated from *Mertk* ^+/+^ (n = 4) and *Mertk* ^−/−^ (n = 2) mice and were cultured for 3 days to expand them prior to RNA isolation. Error bars are standard error of mean (SEM). (**B)** Representative images of isolated Sertoli cells from *Mertk* ^−/−^ and *Mertk* ^−/−^ *Bai1* ^*Tg*^ mice were stained for BAI1 to confirm surface expression of the *Bai1* ^*Tg*^. (**C**) Mice (8–12 weeks-old) underwent testicular torsion surgery to induce ischemic injury. Testicular cross sections from sham and torsion testes were stained for cleaved caspase 3 (CC3) (black arrowheads). Images are of representative tubule cross sections from matched sham and torsion testes. (**D**) The number of CC3 positive cells per tubule cross section was determined by analyzing the entire testicular cross section. Each mouse is represented by individual data points within the bars. *Mertk* ^+/+^ (n = 5) *Mertk* ^−/−^ (n = 8) *Mertk* ^−/−^ *Bai1* ^*Tg*^ (n = 9). Error bars represent SEM. Statistical analysis was performed with a Wilcoxon rank-sum test. *p < 0.05, ***p < 0.001.

Prior to evaluating apoptotic cell accumulation in the testes, we confirmed that Sertoli cells endogenously express *Mertk*, *Bai1* and components of the BAI1 signaling pathway: *Elmo1, Dock180* and *Rac1* (Fig. 1A)^4^. Importantly, expression of *Bai1*, *Elmo1*, *Dock180* and *Rac1* did not differ between *Mertk* ^+/+^ and *Mertk* ^−/−^ mice (Fig. 1A). In addition, we confirmed that the *Bai1* ^*Tg*^ was expressed by Sertoli cells and that the BAI1-Tg properly localized to the surface of *Mertk* ^−/−^ Sertoli cells (Fig. 1B). To determine whether *Bai1* overexpression could rescue phagocytic deficits in *Mertk* ^−/−^ Sertoli cells, we evaluated apoptotic corpse accumulation in testes that had undergone surgical torsion and those that underwent sham surgery^6,8^. Corpse accumulation was quantified by counting cleaved-caspase 3 positive cells in testicular cross-sections (Figs 1C, S1). As was previously reported, *Mertk* ^−/−^ mice exhibited a slight but significant increase in apoptotic corpse accumulation at baseline (Fig. 1C,D **)** ^10^. While the *Mertk* ^−/−^ *Bai1* ^*Tg*^ mice trended towards fewer apoptotic corpses at baseline, this was not statistically significant (Fig. 1D). Analysis of injured testes revealed that *Mertk* ^−/−^ mice had substantially more apoptotic cell accumulation than control littermates (Fig. 1C,D). Interestingly, corpse accumulation in *Mertk* ^−/−^ *Bai1* ^*Tg*^ mice was significantly decreased compared to *Mertk* ^−/−^ mice. In fact, the corpse numbers in *Mertk* ^−/−^ *Bai1* ^*Tg*^ mice were reduced to the number in *Mertk* ^+/+^ mice (Fig. 1C,D). These data suggest that in the context of testicular torsion, overexpression of *Bai1* can reduce the number of apoptotic corpses that seem to accumulate in *Mertk* ^−/−^ Sertoli cells.

### RPE express the *Bai1*^*Tg*^ and BAI1 signaling pathway in *Mertk*^−/−^*Bai1*^*Tg*^ mice

**Figure 2:**
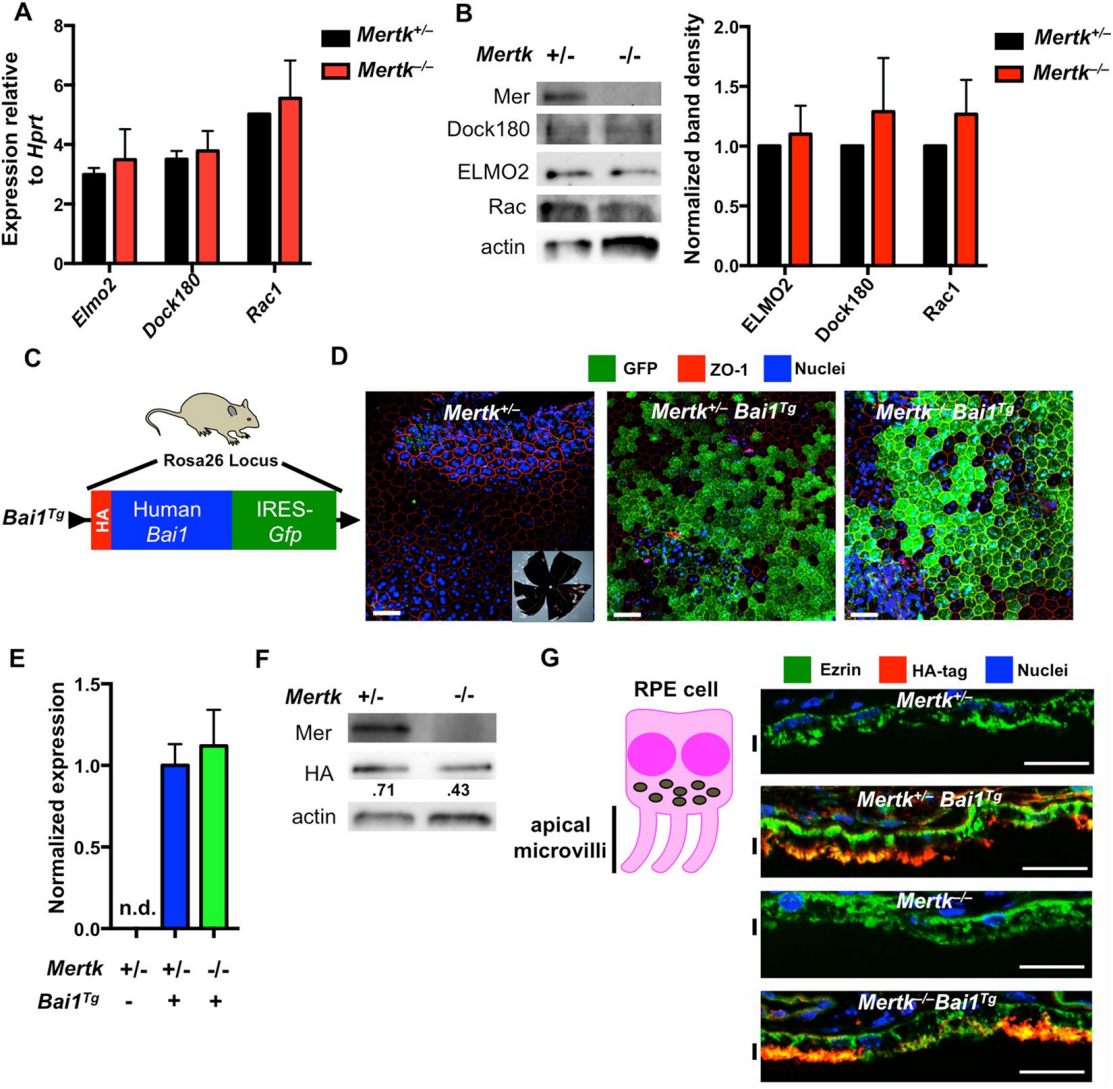
RPE express components of BAI1-signaling pathway and *Bai1* ^*Tg*^. (**A**) Expression of the BAI1 signaling pathway was analyzed by quantitative RT-PCR in RPE isolated from P14 *Mertk* ^+/−^ and *Mertk* ^−/−^ mice (n = 2) Error bars represent SEM. (**B**) Immunoblot analysis of BAI1 signaling pathway in RPE whole cell lysates isolated from P14 mice. Left panel shows representative immunoblots for Mer, Dock180, Elmo2 and Rac. Right panel shows combined densitometry analysis of immunoblots from n = 3 mice per genotype. For densitometry analysis, band volumes were normalized to an actin loading control and band densities in *Mertk* ^−/−^ were then normalized to *Mertk* ^+/−^ for comparison across multiple blots. (**C**) Schematic of *Bai1* ^*Tg*^ insertion in the Rosa26 locus indicating the N-terminal HA-tag in red and IRES-GFP in green. Mouse image licensed from Motifolio Inc. (**D**) GFP expression was analyzed in RPE flat mount preparations at 20x magnification. Inset image in the left panel shows a representative flat mount at 1.2x magnification. White scale bars in images are 50 μm. Images are representative of n = 2 mice per genotype. (**E**) qRT-PCR analysis of *Bai1* ^*Tg*^ expression in *Mertk* ^+/−^ *Bai1* ^*Tg*^ (n = 9) and *Mertk* ^−/−^ *Bai1* ^*Tg*^ (n = 3) RPE isolated on P14. n.d. = not detected. Error bars represent SEM. (**F**) Immunoblot analysis of HA-tag (*Bai1* ^*Tg*^) in RPE whole cell lysates isolated from P13-P14 mice. Immunoblot shown is representative of n = 2 experiments. (**G**) HA staining and localization was analyzed in eyecups at 40x magnification. Neural retinas were removed prior to fixation and staining. Black lines next to the figure align with apical microvilli of RPE. Scale bars are 20 μm. Images shown are representative of n = 2 mice per genotype.

Previous studies have established that POS expose PtdSer that is subsequently recognized by the RPE^13^ and *Mertk* ^−/−^ mice exhibit profound retinal degeneration linked to failed clearance of POS by the RPE^14,15^. While *Mertk* is highly expressed by the RPE^14,15,21^, RPE do not express BAI1 endogenously (Figure S2A). We first confirmed that downstream components of the BAI1 signaling pathway are present within the RPE. Isolated RPE from *Mertk* ^+/−^ and *Mertk* ^−/−^ mice were analyzed by RT-PCR and immunoblotting. These analyses showed expression of the BAI1 signaling components ELMO, Dock180 and Rac1 by RT-PCR (Fig. 2A,B). Importantly, we found that ELMO2, Dock180 and Rac1 were all expressed. Furthermore expression of BAI1 signaling components did not differ between RPE from *Mertk* ^+/−^ and *Mertk* ^−/−^ mice as assessed by both RNA and protein (Fig. 2A,B), suggesting that BAI1 could theoretically function in the RPE.

We next assessed *Bai1* ^*Tg*^ expression and localization within the RPE. The *Bai1* ^*Tg*^ mouse construct contains three separate methods to detect expression *in situ* (Fig. 2C **)**. First, the *Bai1* ^*Tg*^ construct includes an IRES-driven GFP, which allows visualization of the transcriptional activity at the *Bai1* ^*Tg*^ locus. To assess GFP expression in RPE, we prepared RPE flat mounts from *Mertk* ^+/−^ *Bai1* ^*Tg*^ and *Mertk* ^−/−^ *Bai1* ^*Tg*^ mice and imaged GFP by confocal microscopy (Fig. 2D). The GFP expression pattern appears uniform across the RPE layer in both *Mertk* ^+/−^ *Bai1* ^*Tg*^ and *Mertk* ^−/−^ *Bai1* ^*Tg*^ mice. On average, 45% of the area in each 20x field is GFP positive (data not shown). Importantly, no GFP signal was detected in mice lacking the *Bai1* ^*Tg*^ (Fig. 2D). Second, the *Bai1* ^*Tg*^ is derived from the human *Bai1* cDNA^1^. Despite being highly homologous to mouse BAI1 at the protein level and indistinguishable in functional assays^14,18^, human *Bai1* transcript can be distinguished from the murine transcript by RT-PCR. Human *Bai1* was readily detected in the RPE of *Mertk* ^+/−^ *Bai1* ^*Tg*^ and *Mertk* ^−/−^ *Bai1* ^*Tg*^ but not littermate control mice (Fig. 2E). Third, the *Bai1* ^*Tg*^ construct has an N-terminal HA-tag, which facilitates detection of the *Bai1* ^*Tg*^ protein. The HA-BAI1 protein was readily detected in the extracts of isolated RPE cells by immunoblotting (Fig. 2F).

RPE cells are highly polarized and the apical microvilli mediate the phagocytosis of photoreceptor outer segments. Therefore, to assess whether the *Bai1* ^*Tg*^ protein was properly localized on the apical surface in a location similar to MerTK^21^, we stained for the HA-tag in eyecup cross sections. HA-BAI1 showed apical localization and co-localized with ezrin, a cytoskeletal protein enriched in microvilli (Fig. 2G). Furthermore, HA-BAI1 was detected on the apical RPE surface of both *Mertk* ^+/−^ *Bai1* ^*Tg*^ and *Mertk* ^−/−^ *Bai1* ^*Tg*^ mice. These data suggested that RPE cells in the *Mertk* ^−/−^ *Bai1* ^*Tg*^ mice express the *Bai1* ^*Tg*^, and that the BAI1 protein localizes to the region of the RPE that mediates phagocytosis.

To examine whether there are signals initiated by the BAI1 transgene within the RPE, we performed RNAseq on *Mertk* ^+/+^ and *Mertk* ^+/+^ *Bai1* ^*Tg*^ mice to identify genes likely altered by the *Bai1* ^*Tg*^. Significant transcriptional changes were noted in 63 genes from the *Bai1* ^*Tg*^ mice. When we focused on processes previously associated with BAI1, such as cholesterol homeostasis, axonal growth and synaptogenesis^33–35^, we identified 10 genes with functions that are associated with the aforementioned processes (Figure S2B). These data suggest that BAI1 is not only expressed by RPE cells at the correct location, but can also signal to induce transcriptional changes in RPE cells.

### Mertk-linked retinal degeneration is not rescued by BAI1

**Figure 3:**
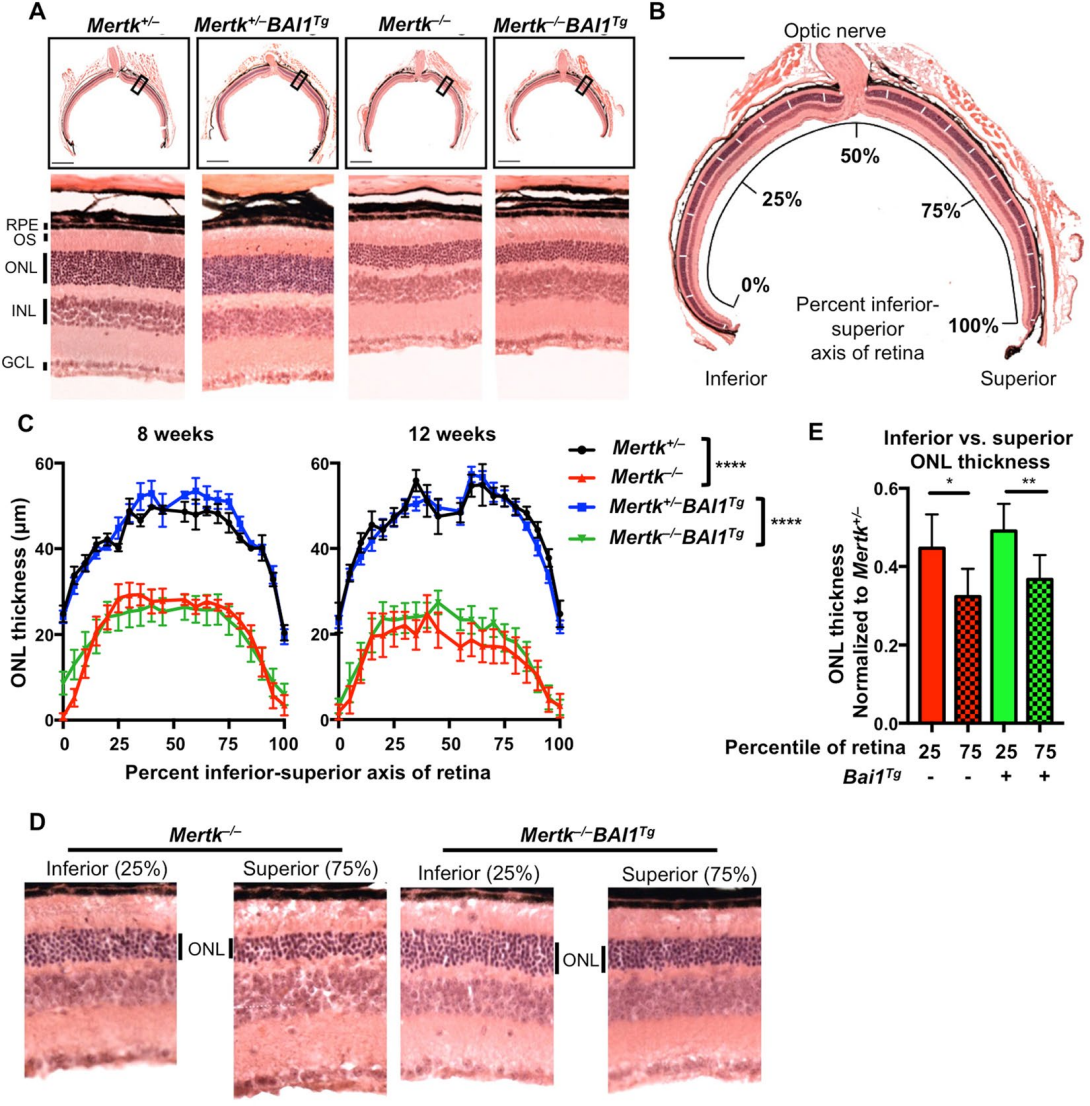
*Bai1* ^*Tg*^ does not rescue retinal degeneration in *Mertk* ^−/−^ mice. (**A**) H&E stained eyecup sections that transect the optic cup were imaged from the different genotypes at 20x magnification. (**B**) An image mask was applied to the images in Photoshop marking the regions to be measured (white dashes overlaid on ONL). Image is a representative eyecup with the overlaid image mask. Scale bar is 500 μm. (**C**) ONL measurements were taken at the indicated points (according to the image mask) along the inferior-superior axis of the retina. The left panel includes measurements from 8 week-old *Mertk* ^+/−^ (n = 3) *Mertk* ^+/−^ *Bai1* ^*Tg*^ (n = 5), *Mertk* ^−/−^ (n = 10) and *Mertk* ^−/−^ *Bai1* ^*Tg*^ (n = 13) mice. Right panel shows measurements from 12 week-old *Mertk* ^+/−^ (n = 6) *Mertk* ^+/−^ *Bai1* ^*Tg*^ (n = 8), *Mertk* ^−/−^ (n = 6) and *Mertk* ^−/−^ *Bai1* ^*Tg*^ (n = 10) mice. Statistical analysis by 2-way ANOVA showed no difference between *Mertk* ^+/−^ and *Mertk* ^+/−^ *Bai1* ^*Tg*^ mice or *Mertk* ^−/−^ and *Mertk* ^−/−^ *Bai1* ^*Tg*^ mice at any time point. Asterisks indicate the difference between genotypes as measured by 2-way ANOVA. Error bars represent SEM. ****p < 0.0001. (**D**) Images of the ONL in the inferior retina (25% measurement mark) and the superior retina (75% measurement mark) from representative mice. (**E**) Quantification of the difference in ONL thickness in inferior retina (25% measurement mark) and the superior retina (75% measurement mark) at 12 weeks of age. *Mertk* ^−/−^ (n = 6) *Mertk* ^−/−^ *Bai1* ^*Tg*^ (n = 10). Significance was determined using a Wilcoxon rank-sum test. *p < 0.05, **p < 0.01. Error bars represent SEM.

We next assessed the retinal degeneration in *Mertk* ^−/−^ *Bai1* ^*Tg*^ mice. Photoreceptors in *Mertk* ^−/−^ mice begin to show signs of overgrowth at post-natal day 17 and exhibit highly disorganized POS by 35 days after birth^14,21^. Degeneration of the photoreceptor layer begins soon afterwards and previous reports have demonstrated that most photoreceptors are lost by 12 weeks of age^14,21,26^. To assess retinal degeneration, we collected sagittal sections of eyes that transect the optic cup (where the optic nerve meets the retina) (Fig. 3A,B). Inspection of central retinas from 12 week-old *Mertk* ^−/−^ and *Mertk* ^−/−^ *Bai1* ^*Tg*^ revealed equivalent degeneration of the outer nuclear layer (ONL) consisting of photoreceptor nuclei. Importantly, *Mertk* ^+/−^ *Bai1* ^*Tg*^ retinal cross sections exhibited normal retinal architecture suggesting that the retinal structure was not adversely affected by transgenic *Bai1* expression (Fig. 2A).

ONL thickness is not equivalent across the retina and degeneration is not necessarily homogeneous^26,27^. To standardize the measurement of ONL across the entire section, we adapted a previously described technique^26^ and measured the ONL thickness at 20 standardized points across the ‘inferior-superior’ axis of the retinal section (Fig. 3B). We measured ONL thickness in mice at 8 and 12 weeks of age (Fig. 3C). At 8 weeks of age, both *Mertk* ^−/−^ and *Mertk* ^−/−^ *Bai1* ^*Tg*^ mice exhibited moderate degeneration across the entire inferior-superior axis (Fig. 3C). By 12 weeks of age, retinal degeneration had progressed in both *Mertk* ^−/−^ and *Mertk* ^−/−^ *Bai1* ^*Tg*^ mice (Fig. 3C). As previously reported^26,27^, degeneration was more severe in the superior than the inferior retina (Fig. 3D,E). Importantly, degeneration was equivalent between *Mertk* ^−/−^ and *Mertk* ^−/−^ *Bai1* ^*Tg*^ animals. Collectively, these data indicate that overexpression of *Bai1* did not rescue retinal degeneration due to loss of *Mertk*.

For analysis of ONL degeneration we facilitated littermate comparison by using *Mertk* ^+/−^ and *Mertk* ^−/−^mice as previous studies demonstrated that the retinas of *Mertk* ^+/−^ mice are indistinguishable from *Mertk* ^+/+^ mice^21,26^. Importantly, we also confirmed that *Mertk* ^+/−^ mice exhibited no retinal degeneration as late as 16 weeks of age (Figure S3A), despite decreased expression of MerTK in *Mertk* ^+/−^ relative to *Mertk* ^+/+^ animals (Figure S3B,C **)**.

### Phagocytosis defect in MerTK-null mice is not rescued by the BAI1 transgene

**Figure 4:**
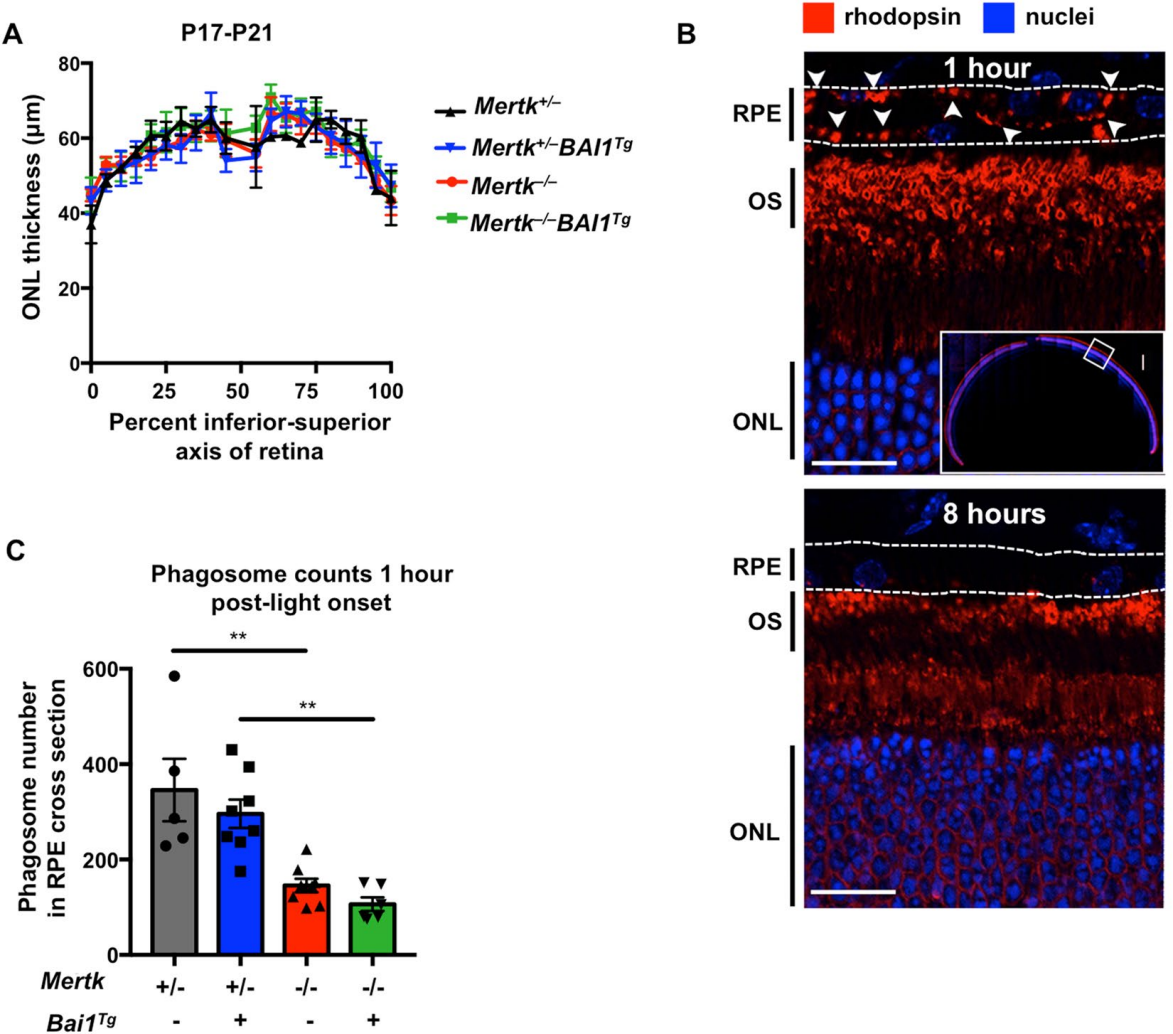
*Bai1* ^*Tg*^ is unable to restore RPE phagocytosis. (**A**) Eyes were isolated from P17-P21 mice 1 h after light onset. ONL measurements were completed as described above. Measurements were taken along the inferior-superior axis at the indicated points from *Mertk* ^+/−^ (n = 2), *Mertk* ^+/−^ *Bai1* ^*Tg*^ (n = 5), *Mertk* ^−/−^ (n = 5), and *Mertk* ^−/−^ *Bai1* ^*Tg*^ (n = 4) mice. Error bars represent SEM. No significant difference was found between any genotypes by 2-way ANOVA. (**B**) Eyecups isolated 1 h after light onset (top panel) were stained for rhodopsin and nuclei (Hoechst). The entire eyecup was imaged at 40x (top panel, inset). Rhodopsin staining is robust in the outer segments (OS) of the photoreceptors. Rhodopsin puncta are apparent in the RPE layer (white arrowheads). Bottom panel shows representative staining from an eyecup isolated 8 h after light onset. (**C**) Rhodopsin puncta in the RPE, referred to as phagosomes, were quantified in ImageJ by an automated particle count. Particle size was constrained from 0.5 μm to ∞ and minimum circularity was restricted to 0.2. Symbols within the bars represent the average particle count from the left and right eyes of one mouse. *Mertk* ^+/−^ (n = 5) *Mertk* ^+/−^ *Bai1* ^*Tg*^ (n = 8) *Mertk* ^−/−^ (n = 8) *Mertk* ^−/−^ *Bai1* ^*Tg*^ (n = 6). Error bars represent SEM. Significance was determined by one-way ANOVA. Multiple comparison analysis was corrected with a post-hoc Tukey’s test.

Retinal degeneration in *Mertk* ^−/−^ mice is attributed to decreased phagocytosis of POS. Therefore, we analyzed RPE phagocytosis of POS in *Mertk* ^−/−^ and *Mertk* ^−/−^ *Bai1* ^*Tg*^ mice. Phagocytosis by RPE *in vivo* is readily analyzed due to its temporal regulation (around light onset) and because the content of the phagosomes (rhodopsin) can be visualized^14,30^. The rate and amount of phagocytosis is a dual function of phagocyte efficiency and the ratio of targets to phagocytes. Since *Mertk* ^−/−^ mice exhibit photoreceptor degeneration, the number of POS targets decreases as degeneration progresses. Therefore, decreases in RPE phagocytosis after the onset of degeneration could be due to the inefficient uptake by the RPE or a decreased target to phagocyte ratio. To ensure that any observed changes in phagocytosis were due to RPE phagocytic efficiency, we analyzed mice prior to the onset of retinal degeneration, at P17-P21 days of age. Importantly, analysis of ONL thickness at this age confirmed that degeneration in *Mertk* ^−/−^ and *Mertk* ^−/−^ *Bai1* ^*Tg*^ mice had not yet begun (Fig. 4A).

To quantify phagocytosis, we harvested eyes 1 h after light onset and immunostained sagittal-eye sections for rhodopsin (Fig. 4B, white arrows). Importantly, rhodopsin puncta are degraded over time and thus are mostly absent 8 h after light onset, suggesting that these puncta are not artifacts of sectioning or staining (Fig. 4B). Quantification of puncta at 1 h post-light onset revealed a striking decrease in rhodopsin puncta in the RPE of *Mertk* ^−/−^ mice relative to *Mertk* ^+/−^ mice. However, neither *Mertk* ^+/−^ *Bai1* ^*Tg*^ nor *Mertk* ^−/−^ *Bai1* ^*Tg*^ mice exhibited differences in the number of puncta compared to their respective controls (Fig. 4C). Furthermore, neither the loss of *Mertk* nor overexpression of BAI1 affected phagosome trafficking in RPE, as indicated by basolateral localization of phagosomes (Figure S4 **)**. BAI1 overexpression was previously shown to enhance phagocytosis in WT epithelial cells (both *in vitro* and *in vivo*)^1^. However, BAI1 was unable to enhance phagocytosis in *Mertk* ^+/+^ mice and importantly, the number of puncta in *Mertk* ^+/+^ mice were equivalent to the number seen in *Mertk* ^+/−^ (Figure S3D). Collectively, these data suggest that *Bai1* ^*Tg*^ expression is unable to rescue the RPE phagocytic defect observed in *Mertk* ^−/−^ mice.

### MerTK regulates the gene expression program in the RPE

The lack of rescue by BAI1 prompted us to ask whether MerTK regulates the phagocytic capacity of RPE in ways beyond PtdSer binding. To address this question, we chose an unbiased transcriptomics approach, and performed RNAseq analysis of RPE from *Mertk* ^+/+^ and *Mertk* ^−/−^ mice. RPE were isolated at day 14 after birth, a time when the mouse pups have opened their eyes but *Mertk* ^−/−^ mice do not yet exhibit photoreceptor overgrowth that could impact RPE gene expression^14,21^.

**Figure 5:**
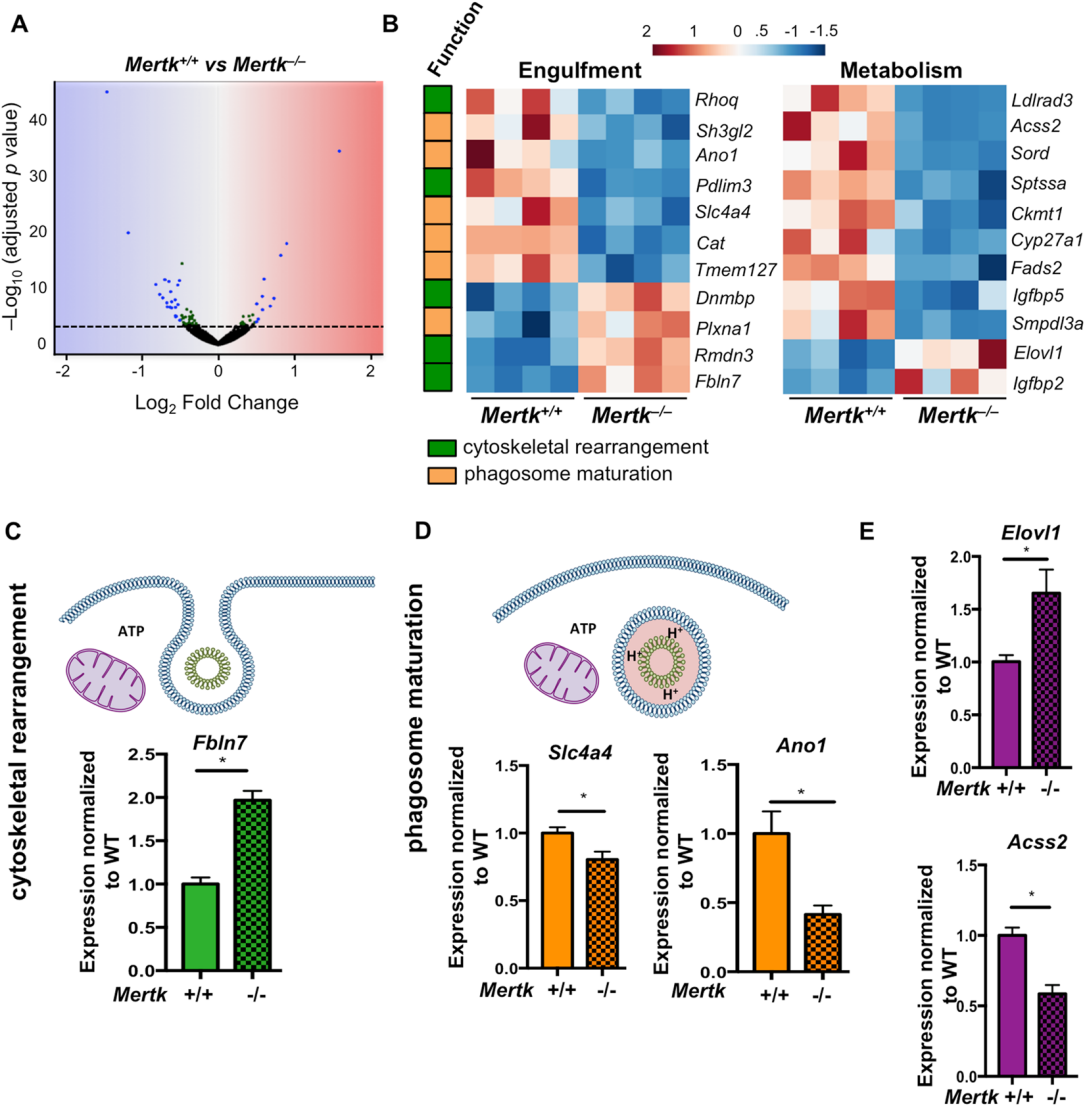
*Mertk* regulates multiple genes linked to phagocytosis and metabolism. (**A**) RNAseq was performed on RPE isolated from P14 *Mertk* ^+/+^ (n = 4) and *Mertk* ^−/−^ (n = 4) mice 2 h after light-onset. DEseq. 2 analysis identified 60 genes that were differentially expressed according to p values adjusted for multiple comparisons. The log_2_ fold change and –Log_10_padj values are plotted for all hits. Green dots represent genes that had a padj value <0.05. Blue dots indicate genes with a padj <0.05 and log_2_ fold change >0.5. (**B**) Further annotation of the differentially regulated genes revealed 11 linked to pathways important in phagocytosis and 11 linked to metabolic pathways. These genes and their expression changes are depicted in heat maps. Functional associations with regard to phagocytosis are indicated on the left. (**C**) Genes related to cytoskeletal reorganization (graphs in green), (**D**) genes related to phagosome maturation (graphs in yellow) and (**E**) genes related to metabolism (graphs in purple) were selected for further validation by qRT-PCR. Validation studies were done with fresh RPE samples from P14 *Mertk* ^+/+^ (n = 4) and *Mertk* ^−/−^ (n = 4) and original library preparations for a total of 8 samples per genotype. Significance was calculated by student’s T-test. *p < 0.05.

RNAseq analysis identified 60 genes that were differentially regulated in *Mertk* ^−/−^ RPE (Fig. 5A). Further analysis revealed that 11 of these genes have annotated functions related to either cytoskeletal rearrangement or endosomal maturation, two processes that are essential for phagocytosis (Fig. 5B). The gene fibulin 7 (*Fbln7*), important for extracellular matrix adhesion and cytoskeletal rearrangement^36–38^, has a SNP associated with age-related macular degeneration and its expression is altered in patients with retinoschisis, a form of inherited retinal dystrophy^39,40^. qRT-PCR validation of the original library and an additional cohort of *Mertk* ^+/+^ and *Mertk* ^−/−^ mice confirmed that *Fbln7* is upregulated in *Mertk* ^−/−^ RPE (Fig. 5C). Phagosome maturation culminates in acidification, which promotes the breakdown of the phagosome content^41^. Chloride flux across the lysosome membrane regulates lysosome acidification^42,43^. Therefore we further assessed the chloride channel anoctamin 1 (*Ano1*) and the bicarbonate transporter *Slc4a4* for further validation. Although *Ano1* has yet to be implicated in the chloride flux across lysosomal membranes, it is a well-established calcium-activated chloride transporter^44^ and RPE phagocytosis is accompanied by substantial calcium accumulation in the RPE^45^. *Slc4a4* is a bicarbonate transporter that is thought to regulate intracellular pH by electrogenic flux of H^+^ and HCO_3_^−^^46^. Interestingly, it has been proposed that these ion transporters work together in certain cellular processes^47^. We validated that *Slc4a4* and *Ano1* were reproducibly downregulated in both the original RPE samples as well as in an additional cohort of RPE (Fig. 5D). These data suggest that the presence of MerTK can regulate the expression of various components of the phagocytic machinery, either directly or indirectly.

Phagocytosis is a metabolically demanding process for the phagocyte that requires significant ATP generation to mediate cytoskeletal rearrangement and digestion of cargo^48,49^. In addition to annotating multiple genes involved in phagocytosis, we identified an additional 11 genes related to cell metabolism (Fig. 5B). Although these genes we identified have yet to be directly linked to phagocytic processes, the number of genes that were differentially regulated was intriguing. We validated the fatty acid elongase, *Elovl1* and the acetyl CoA synthetase, *Acss2*, and found that the expression changes were reproducible, in both the original library and a fresh cohort of *Mertk* ^+/+^ and *Mertk* ^−/−^ mice (Fig. 5E). Overall, these data suggest that MerTK might coordinate the phagocytic process at multiple levels from PtdSer binding, to cytoskeletal reorganization and phagosome maturation, as well as coordinating metabolic changes during phagocytosis (and potentially the visual cycle).

In addition, 9 genes identified from the RNAseq screen were linked to human retinal disease (Table 1). Five of these genes were also annotated under the metabolic subset (marked with*) and three were annotated in the phagocytosis subset (marked with #) (Fig. 5B, Table 1). Two genes, *Gja1* and *Cyp27a1* have a striking monogenic association with oculodentodigital dysplasia and cerebrotendinous xanthomatosis respectively, both of which are associated with retinal abnormalities^50,51^. Three genes had SNP associations to retinal disease: *Ldlrad3*, *Fads2*, and *Fbln7* (Table 1). Five of the genes we annotated as being linked to retinal disease, were identified in the comparative toxicogenomics database, which infers gene-disease associations by examining curated associations between pharmacological agents, known diseases and the gene of interest^52,53^. These associations were further validated by experimental links to retinal disease or RPE function. *Slc4a4* has also been linked to retinitis pigmentosa (RP) type 17 and *Elovl1* is an endogenous inhibitor of the visual cycle enzyme RPE65, defects in which cause RP type 20^54–56^. Overall, our findings suggest that the loss of MerTK (directly or indirectly) perturbs multitude of genes, which might influence additional RPE functions beyond phagocytic pruning of photoreceptors.

**Table 1:** The absence of *Mertk* alters expression of genes linked to human retinal diseases.

Gene	Log_2_ fold change	Reported retinal disease association in humans	Experimental evidence
Ldlrad3*	−1.08	SNP^66^, inferred^52,53^	Intronic SNP associated with pathological myopia, a disease associated with degeneration of several eye structures including RPE^67–69^
*Gja1*	−0.54	Monogenic^51,70^, inferred^52,53^	Mutations in *Gja1* have a monogenic association with oculodentodigital dysplasia, which has multiple manifestations including retinal dysplasia^51^. *Gja1* is also critical in RPE differentiation and communication between RPE cells^71,72^.
Cyp27a1*	−0.48	Monogenic^50^, inferred^52,53^	Mutations in *Cyp27a1* have a monogenic association with cerebrotendinous xanthamatosis, which is associated with premature retinal senescence^50,73^. Mice with mutations in Cyp27a1 exhibit abnormal retinal vascularization and cholesterol deposits in the RPE^74,75^.
*Slc4a4* ^#^	−0.48	inferred^52–54,76^	Retinitis pigmentosa 17 (RP17) is characterized by mutations in *Ca4* ^77,78^. Some variants of RP17 have *Ca4* mutations that prevent interaction and activation of *Slc4a4* ^54^.
*Cat* ^#^	−0.48	inferred^76,79–81^	Increased expression of *Cat* in RPE prevents oxidative damage to photoreceptors^80^. Age-related macular degeneration (AMD) is associated with decreased catalase activity in RPE^79^.
Fads2*	−0.36	SNP^82^, inferred^52,53^	SNPs in intronic and regulatory regions of *Fads2* have been linked to AMD^82^. *Fads2* ^−/−^ mice exhibit structural changes in interphase between RPE and photoreceptors^83^.
Igfbp5*	−0.36	inferred^52,53^	Altered *Igfbp5* expression is associated with myofibroblastic changes in RPE^84^.
Elovl1*	0.45	inferred^52,53^	ELOVL1 is an endogenous inhibitor of the visual cycle enzyme, RPE65^56^. Mutations in *Rpe65* cause retinitis pigmentosa^55^.
*Fbln7* ^#^	0.82	SNP^39^, inferred^52,53^	Intronic SNP associated with reduced severity of AMD^39^.

*Indicates gene function is associated with metabolism.

^#^Indicates gene function is associated with phagocytosis.

### MerTK^−/−^mice exhibit diminished retinyl ester accumulation prior to the onset of degeneration

**Figure 6:**
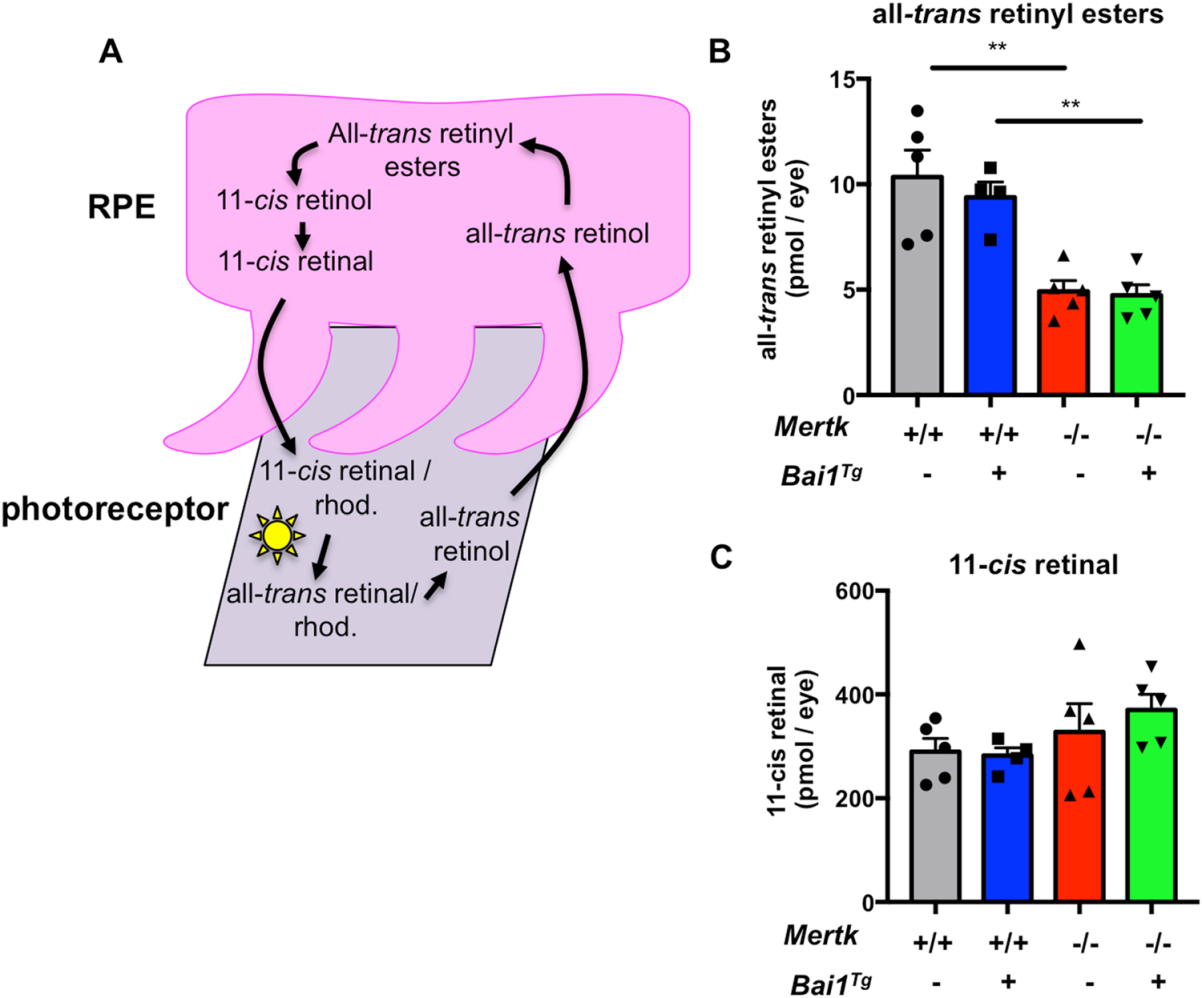
*Mertk* ^−/−^ mice have reduced retinyl ester accumulation. (**A**) Schematic of the visual (retinoid) cycle that occurs in RPE (pink) and POS (gray). (**B**) P20-P21 mice were dark adapted overnight and HPLC analysis was performed to quantify all-*trans*-retinyl esters and (**C**) 11-*cis-*retinal. Individual points within bars are the average value for both eyes from one mouse: *Mertk* ^+/+^(n = 5) *Mertk* ^+/+^ *Bai1* ^*Tg*^ (n = 4) *Mertk* ^−/−^ (n = 5) *Mertk* ^−/−^ *Bai1* ^*Tg*^ (n = 5). Error bars represent SEM. Statistical analysis was performed by one-way ANOVA. Multiple comparisons were corrected with a post-hoc Tukey’s test. **p < 0.01.

In addition to promoting photoreceptor survival, RPE contribute indirectly to phototransduction by participating in the visual cycle, which supplies photoreceptors with the chromophore 11-*cis*-retinal. The visual or retinoid cycle is the recycling of vitamin A (all-*trans-*retinol) to the light-reactive chromophore 11-*cis*-retinal (Fig. 6A)^32^. Interestingly, one of the first characterizations of *Mertk* ^−/−^ mice revealed that vision is affected in mice as early as P20, prior to the onset of retinal degeneration^14^. Given the broader changes in gene expression that we observed in *Mertk* ^−/−^ RPE, we investigated whether *Mertk* expression influenced the visual cycle.

High-performance liquid chromatography was used in a blinded assay to quantify all-*trans-*retinyl esters and 11-*cis*-retinal in the eyes of dark-adapted mice at P21 (prior to degeneration but after significant retinal maturation)^14,21,57,58^. Retinyl esters are notoriously difficult to extract and quantify, thus we developed a novel procedure that allows extraction and quantification of the esters with more than 90 percent accuracy^59^. Interestingly, we found that all-*trans-*retinyl ester levels were significantly lower in *Mertk* ^−/−^ relative to WT mice (Fig. 6B). However, there was no difference in the levels of the chromophore 11-*cis*-retinal at this time point (Fig. 6C). Retinyl esters are a form of stored vitamin A; esterification to fatty acids prevents their release in to the extracellular space^60,61^. The shifts we observed in metabolic gene expression could influence the availability of fatty acids for esterification, possibly affecting retinyl ester accumulation. Diminished retinyl ester levels in *Mertk* ^−/−^ mice prior to the loss of photoreceptors support the hypothesis that *Mertk* can influence critical processes beyond phagocytosis, and that MerTK is uniquely critical for RPE function.

## Discussion

Phagocytes express multiple PtdSer receptors and it has been unclear whether these receptors are functionally redundant or if they have tissue and context specific functions. In an attempt to determine if PtdSer receptors are functionally unique, we asked if PtdSer receptors are capable of compensating for one another in specialized phagocytes *in vivo*. Our analysis of the *Mertk* ^−/−^ *Bai1* ^*Tg*^ mice led to two major findings. First, *Bai1* ^*Tg*^ expression appears to rescue phagocytic deficits in *Mertk* ^−/−^ Sertoli cells. Second, *Bai1* ^*Tg*^ expression was not able to prevent retinal degeneration or rescue phagocytic deficits in the *Mertk* ^−/−^ RPE. These findings provide the first evidence for functional compensation between two different PtdSer phagocytosis receptors from two distinct receptor families: The adhesion family GPCR, BAI1 and the receptor tyrosine kinase MerTK. Our results also suggest that the role of a given PtdSer receptor in cell clearance may differ depending on the tissue context. Overall, this indicates that PtdSer receptors are not fully interchangeable, which could, in part, explain why phagocytes express a variety of PtdSer receptors on their cell surface. It also suggests that expression of MerTK is non-redundant in the eye/retina.

To understand what unique role(s) MerTK might have in the RPE, we performed RNAseq on *Mertk* ^−/−^ and *Mertk* ^+/+^ RPE and identified 60 genes that were differentially expressed in *Mertk* ^−/−^ RPE cells. From those genes, three critical subsets were dysregulated: genes linked to retinal disease, phagocytosis, and metabolism. Genes linked to human retinal disease have associations that are either monogenic, SNP, or inferred based on curated experimental and pharmacological data. Interestingly, 8 of the 9 genes associated with retinal disease were linked to either metabolic or phagocytic functions. These perturbations could explain why *Mertk* ^−/−^ mice exhibit visual defects (as measured by electroretinogram recordings) at P20, prior to the onset of retinal degeneration^14^. Furthermore, the observed transcriptional changes may help to explain the decreased retinyl ester accumulation in *Mertk* ^−/−^ mice as our data revealed significant changes in metabolic genes that might perturb the availability of fatty acids for esterification.

The finding that *Bai1* overexpression can reduce apoptotic corpse accumulation in the testes is intriguing. Though RPE phagocytosis of POS is PtdSer dependent, it is not ‘traditional apoptotic corpse clearance’ and could instead be analogous to the cell pruning observed in the nervous system. It is possible that the mechanism of action of BAI1 in phagocytosis is not conducive to pruning-type events, but rather that BAI1 is more suited for larger corpse uptake. Alternatively, BAI1-mediated phagocytosis could require an as-of-yet unidentified co-factor that RPE cells do not express. This might explain why the *Bai1* ^*Tg*^ can enhance phagocytosis in *Mertk* ^−/−^ Sertoli cells, where it is known to function endogenously^1,62^.

Overall, this work suggests that the variety of PtdSer receptors did not evolve as a simple redundancy mechanism. Rather, these receptors likely play unique roles beyond PtdSer recognition, including the regulation of genes crucial to the process of phagocytosis and biological processes essential for general homeostasis and even specialized tissue functions. Overall, this suggests that PtdSer receptors should not be regarded as having homogenous functionality. Further research into the downstream functions of PtdSer receptors is critical to understanding how the phagocytic process is coordinated in different tissues and contexts.

### Experimental Procedures

#### Mice

*Mertk* ^−/−^ mice were purchased from Jackson Laboratories (stock no: 011122 – B6;129-*Mertk* ^tm1Grl^/J). *Bai1* ^*Tg*^ mice were previously generated by our lab on a C57Bl/6 N background and then backcrossed with C57Bl/6 J mice^1^. The *Bai1* ^*Tg*^ mice were screened for the RD8 mutation and were confirmed to be RD8 negative^63^ *Mertk* ^−/−^ mice were crossed to *Bai1* ^*Tg*^ mice to generate the first generation of *Mertk* ^+/−^ *Bai1* ^*Tg*^ mice. Progeny from this initial cross were bred to the original *Mertk* ^−/−^ line from Jackson Laboratories. For line maintenance, *Mertk* ^+/−^ *Bai1* ^*Tg*^ progeny were crossed to the original *Mertk* ^−/−^ line from Jackson Laboratories. *Mertk* ^+/+^ and *Mertk* ^+/+^ *Bai1* ^*Tg*^ mice were generated by crossing *Mertk* ^+/−^ and *Mertk* ^+/−^ *Bai1* ^*Tg*^ siblings. Mice were maintained on a 14–10 h light-dark cycle. Animals for analysis were euthanized by CO_2_ asphyxiation. All animal procedures were approved by and performed according to guidelines of the Institutional Animal Care and Use Committee (IACUC) at the University of Virginia.

#### RPE isolation for gene expression analyses

The RPE isolation protocol was previously described^64^. Eyes were enucleated from P14 neonatal mice 2 hours after light onset. Globes were incubated in serum free DMEM (Corning) with 2% dispase (Worthington). Following incubation, eyes were washed 3 times in DMEM supplemented with 10% fetal bovine serum (FBS) (Gemini). Cornea, iris and lens were removed from each eyecup. The eyecups were incubated for 20 minutes at 37 °C in DMEM supplemented with 10% FBS. Following incubation, the neural retina was removed from each eyecup. The eyecup was tugged at opposite ends to release the RPE layer. RPE sheets were washed in Ca^2+^/Mg^2+^ free HBSS (Gibco). RPE to be used for RT-PCR or RNAseq were lysed in 350 μL of RLT buffer (Qiagen). RPE to be used for protein analysis were lysed in 40 μL of RIPA buffer.

#### Immunoblotting

Crude RPE lysates in RIPA buffer were sonicated 2 times for 10 s to shear DNA. Sonicated lysates were incubated at 37 °C in Laemmli sample buffer for 30 min. Lysates were run on Any-kD stain free gels (Bio-Rad) and transferred to PVDF membranes. Membranes were blocked in Tris buffered saline with Tween 20 (TBS-T) with 5% milk for 1 h at room temperature. The following primary antibodies were used at 1:1000 dilution unless otherwise indicated: MerTK (R&D #AF591), HA (Cell Signaling Technology, clone C29F4), Rac1 (Millipore, clone 23A8), Dock180 1:200 (Santa Cruz #6043 and #6167), ELMO2 (in-house^65^), and β-actin 1:100,000 (Sigma clone AC-15).

#### RT-PCR

RNA was purified from cell lysates with RNeasy kit (Qiagen). cDNA was prepared with Superscript III kit (Thermo Fisher). The following Taqman probes (Thermo Fisher) were used for qPCR: *Bai1* (Mm00558144_m1), *Bai1* human (Hs01105174_m1), *Bai2 (*Mm00557365_m1), *Bai3* (Mm00657451_m1), *Mertk* (Mm00434920_m1), *Dock1* (Mm01269874_m1), *Elmo2* (Mm01248046_m1) *Elmo1* (Mm00519109_m1), *Rac1* (Mm01201657_m1), *Ano1* (Mm00724407_m1), *Elovl1* (Mm01188316_g1), *Acss2* (Mm00480101_m1), *Fbln7* (Mm01336227_m1) *β2* *m* (Mm00437762_m1), *Hprt* (Mm00446968_m1), and *Slc4a4 (*Mm01347935_m1).

#### Eyecup dissection and ONL analysis

Central corneas of enucleated eyes were punctured with a 25-gauge needle. Eyes were submerged in Hartman’s fixative (Sigma) and incubated for 1–3 h at room temperature. Following initial fixation, the cornea, iris and lens were removed. Eyecups were returned to fixative and incubated overnight at 4 °C. Eyecups were moved to 30% sucrose for cryo-protection and incubated at 4 °C until they sank. Eyecups were embedded in O.C.T. (Tissue-Tek) and flash frozen in an isobutane dry ice bath. Consistent orientation of the ‘nasal-notch’ during embedding was maintained to control eyecup orientation. Sagittal eyecup sections were cut at 10 μm thickness and sections transecting the optic cup were collected. Sections for ONL analysis were stained with Mayer’s haematoxylin (Sigma) and eosin (Fisher) and tiled at 20x magnification on an Axio Imager.z1 (Zeiss) with Stereo Investigator software (MBF Biosciences). An image mask with 20 fixed measurement points was applied to eyecup images in Photoshop (Adobe). The ONL was measured at points indicated by the mask in ImageJ software (NIH).

#### *In situ* rhodopsin analysis

P17-P21 mice were euthanized 1 h after light onset. Eyes were dissected, fixed and sectioned as described above. Eye sections were blocked in PBS (Corning) with 10% normal goat serum (Jackson Immunoresearch) for 1 h at room temperature. Sections were stained overnight with rhodopsin antibody diluted 1:500 (Abcam clone Rho 4D2). AF647 conjugated secondary antibody was used to detect rhodopsin. Tiled images were acquired at 40x magnification on an Axio Observer.z1 microscope (Zeiss). The RPE layer was isolated in Photoshop (Adobe). Quantification of puncta was performed by automated particle count in ImageJ (NIH).

#### Flat mount preparation and staining

Mice were euthanized between 4 and 6 weeks of age. Eyes were enucleated and the cornea, iris, lens and neural retina were removed. Eyes were cut at four points for flat mount ‘clover’ preparation and fixed for 1 h in PBS 4% paraformaldehyde (PFA) at room temperature. Flat mounts were blocked and permeabilized in PBS 10% normal horse serum (Hyclone) and 0.1% Tween 20 then stained over night at 4 °C with anti-GFP antibody at 1:50 (Abcam #6673). Secondary antibody conjugated to AF488 was used to detect GFP staining. Flat mounts were then stained with anti-ZO-1 at 1:250 (Thermo Fisher #61-7300) for 1 h at room temperature. Secondary antibody conjugated to AF647 was used to detect ZO-1 staining. Images were acquired at 20x magnification on a confocal Axio Observer.z1 microscope (Zeiss).

#### Eyecup staining for HA

Mice were euthanized between 4 and 6 weeks of age. Eyes were enucleated and the cornea, iris, lens, and neural retinas were removed. Eyecups were fixed for 1 h in PBS 4% PFA at room temperature then moved to 30% sucrose for cryo-protection and incubated at 4 °C until the eyecups sank. Eyes were embedded in O.C.T. Eyecups were sectioned at 10 μm and sections bisecting the optic cup were collected. Sections were blocked in PBS with 10% normal goat serum for 1 h at room temperature. Sections were stained overnight at 4 °C with anti-HA antibody 1:100 (Cell Signaling Technology clone C29F4). AF647 conjugated secondary antibody was used to detect staining (ThermoFisher). Sections were then stained with biotin conjugated anti-ezrin antibody 1:100 (ThermoFisher clone 3C12) overnight at 4 °C. AF488 conjugated streptavidin was used to detect ezrin staining (ThermoFisher). Images were acquired at 40x magnification on an Axio Imager.z2 microscope with Apotome (Zeiss).

#### Testicular torsion

Torsion surgery was performed as previously described^8^. The studies were performed in accordance with the ‘Guiding Principles of the Care and Use of Research Animals’ promulgated by the Society for the Study of Reproduction. The male mice were anesthetized with an intraperitoneal (IP) injection of a mixture of 6 mg /100 g of ketamine and 0.5 mg/100 g of xylazine. After the testes were exteriorized through a low ventral midline incision, the testes were released from the epididymo-testicular membrane through incising the gubernaculums. For torsion, the testis of right side was rotated 720° for 2 h, during which time it remained in the abdomen with a closed incision. For sham control, the testis was freed of the epididymo-testicular membrane and left in the abdomen. After 2 h, the incision was reopened, the testis was counter-rotated to the natural position, the gubernaculum was rejoined, the testes were reinserted into the scrotum, and then the incision was closed. 24 h after operation, mice were euthanized. The testes were removed and fixed for 6 h in Bouin’s fixative for paraffin embedding. Testicular cross sections were stained for cleaved caspase-3 at the University of Virginia Biorepository and Tissue Research Facility. Testicular cross sections were imaged at 20x with an Aperio Scanscope at the University of Virginia Biomolecular Analysis Facility and Shared Instrumentation Core. The number of apoptotic cells per tubule was determined for the entire cross section.

#### Sertoli cell isolation and staining

Testes from P11-P21 day-old mice were isolated and decapsulated. Tubules were dispersed in a solution of HBSS, 0.0625% Trypsin (Corning) 10 μg/mL DNase (Sigma) for 20 min. at 37 °C then 0.00625% Soybean trypsin inhibitor (SBTI) (Sigma) was added and the supernatants were decanted. Tubules were re-suspended in HBSS with 1 M glycine, 2 mM EDTA, 0.0625% SBTI, and 10 μg/mL DNase and incubated for 10 min. at room temperature. Suspensions were spun at 1000 RPM. Tubules were minced and re-suspended in HBSS with 1 mg/mL collagenase and 10 μg/mL DNase in a shaking water bath at 37 °C for 30 minutes. Tubule fragments were allowed to sediment at room temperature and re-suspended in HBSS with 15 U/mL hyaluronidase (Sigma) and 10 μg/mL DNase and incubated in a shaking water bath at 37 °C for 30 min. Tubule fragments were centrifuged at 1000 RPM. Pellets are washed and re-suspended in F12/DMEM (Corning) with 10% FBS, 1% PSQ 1% (Corning), sodium pyruvate (Corning), 10 mM HEPES (Corning), 5 μg/mL Transferrin (Sigma), 2.5 ng/mL epidermal growth factor (Gibco), 10 μg/mL insulin (Gibco). Media was changed after 24 h of culture to remove floating germ cells. Sertoli cell cultures were passaged after 3–5 days to chamber slides for staining. Sertoli cells were fixed in PBS with 4% PFA for 30 min. at room temperature, blocked in PBS with 10% normal goat serum (Jackson Immunoresearch) and stained overnight in anti-BAI1 antibody 1:100 (R&D #AF4969).

#### Retinoid analysis

P20-P21 mice were dark adapted overnight. Eyes were collected under dim red light and stored at −80 °C until they were analyzed. Whole globes were then homogenized in 10 mM sodium phosphate buffer, pH 8.0, containing 50% methanol (v/v) and 100 mM hydroxylamine. The resulting mixture was extracted twice with 4 ml of ethyl acetate. The combined organic layers were dried in vacuo, reconstituted in 400 μl of hexanes, and 100 μl of the extract was injected on to a normal-phase high-performance liquid chromatography (HPLC) (Agilent Sil, 5 *μ*m, 4.6 × 250 mm; Agilent Technologies, Santa Clara, CA) in a stepwise gradient of ethyl acetate in hexanes (0–17 min, 0.6%; 17.01–42 min. 10%) at a flow rate of 1.4 ml·min^−1^. Retinoids were detected by monitoring absorbance at 325 nm and quantified based on a standard curve representing the relationship between the amount of synthetic retinoid standard and the area under the corresponding chromatographic peak.

#### RNAseq

RPE were isolated from littermate P14 mice 2 h after light onset. RNA was isolated with an RNeasy kit (Qiagen). An mRNA library was prepared using an Illumina TruSeq platform. The transcriptome was sequenced on a NextSeq. 500 cartridge. The statistical software package R (version 3.3.2) was used for all analyses. The Bioconductor package DESeq. 2 was used for differential gene expression analysis of RNA-seq data. Heatmaps were created using the R package gplots via the heatmap.2 package. The R code used for bioinformatics analysis and heatmap generation is available upon request.

#### Statistics

Statistics analysis was performed in GraphPad Prism 7.0. Statistical test used as indicated in the figure legends. A p-value < 0.05 was considered statistically significant.

### Data availability

All data is available upon request. RNAseq datasets are publicly accessible on GEO.

## Electronic supplementary material

supplemental data
