# Supplementary material for: Context-dependent compensation among phosphatidylserine-recognition receptors
Source: Sci Rep. 2017 Nov 7;7:14623. doi: 10.1038/s41598-017-15191-1 (PMC5676788; doi:10.1038/s41598-017-15191-1)
Supplement: Supplementary file 1 — supplemental data [file 41598_2017_15191_MOESM1_ESM.pdf]

Supplementary Figures

Context-dependent compensation among phosphatidylserine-recognition receptors

Kristen K Penberthy, Claudia Rival, Laura S Shankman, Michael H Raymond, Jianye Zhang, Justin S A Perry, Chang Sup Lee, Claudia Z Han, Suna Onengut-Gumuscu, Krzysztof Palczewski, Jeffrey J Lysiak, Kodi S Ravichandran

Figure S1: Testicular cross sections

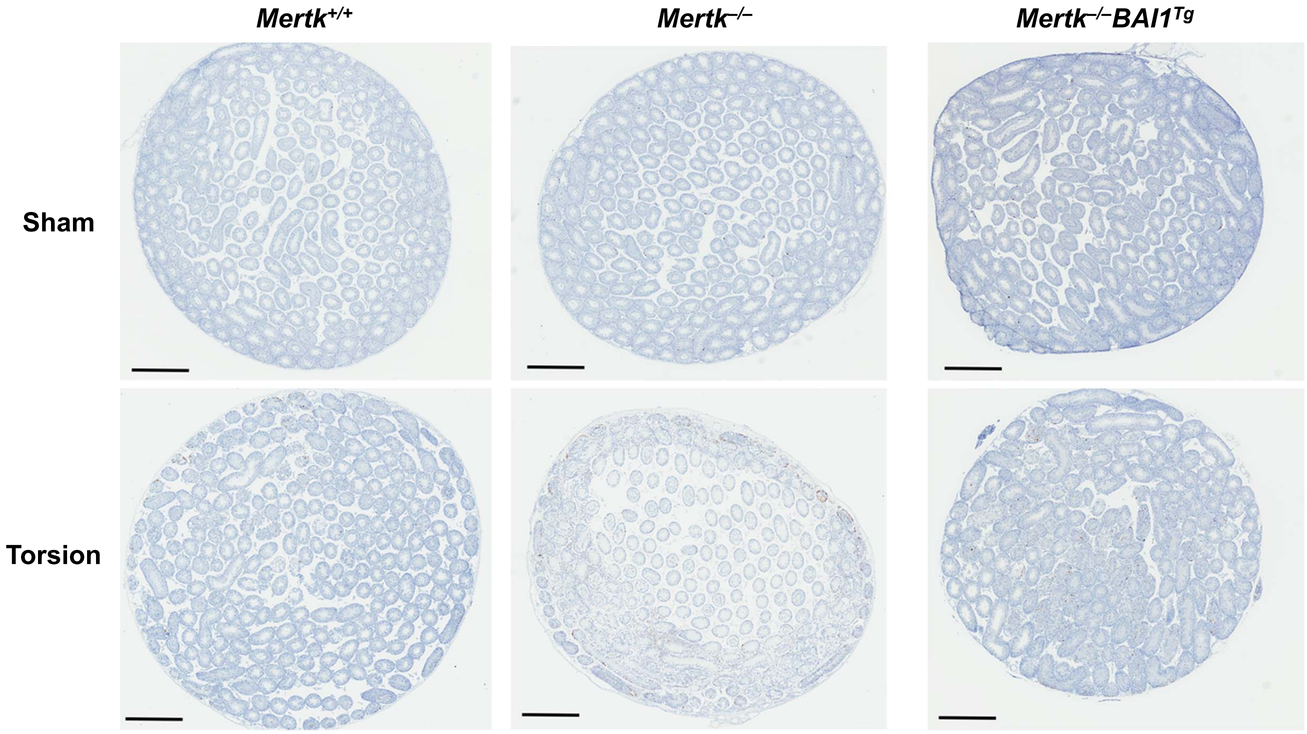

Figure S1: Testicular cross sections
Representative scans of testicular cross sections from sham and torsion testes. Scans were magnified 20x and CC3+ cells were counted. Scale bar = 700 μm.

Figure S2: *Bai1^{Tg} influences gene transcription in RPE despite lack of endogenous Bai1*

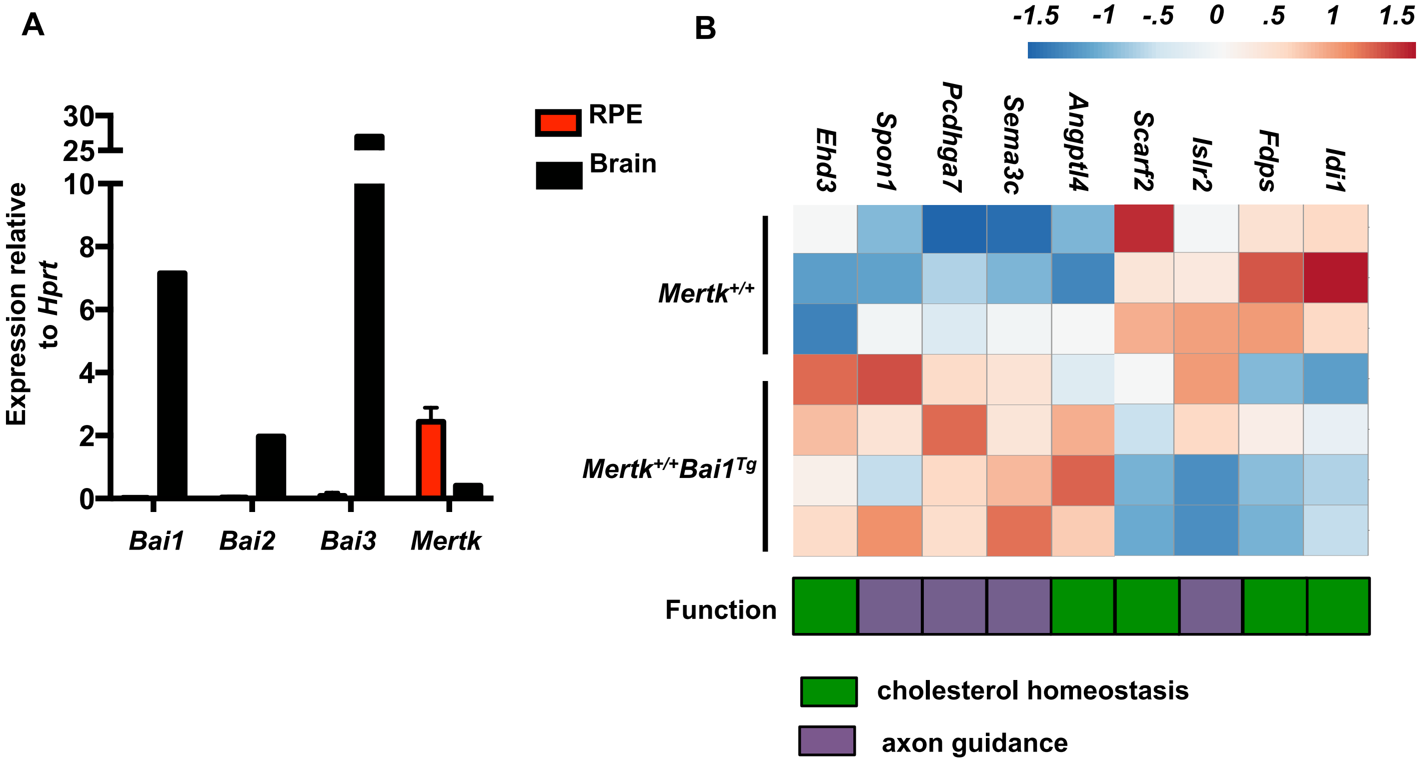

Figure S2: : *Bai1^{Tg} influences gene transcription in RPE despite the lack of endogenous Bai1*

a) RPE expression of *Bai1, Bai2* and *Bai3* was assessed by quantitative RT-PCR in *Mertk$^{+/+}$* mice (n=2). *Mertk* expression was also evaluated for comparison. Cortex was used as a positive control for *Bai* family expression.

b) RNAseq was performed on *Mertk$^{+/+}$*and *Mertk$^{+/+}$Bai1^{Tg}* RPE. Genes related to different BAI1 functions were annotated in the heatmap and annotated gene function is noted on the right.

Figure S3: *Mertk*$^{+/+}$ and *Mertk*$^{+/-}$ mice display no phenotypic differences in retinal degeneration and phagocytosis

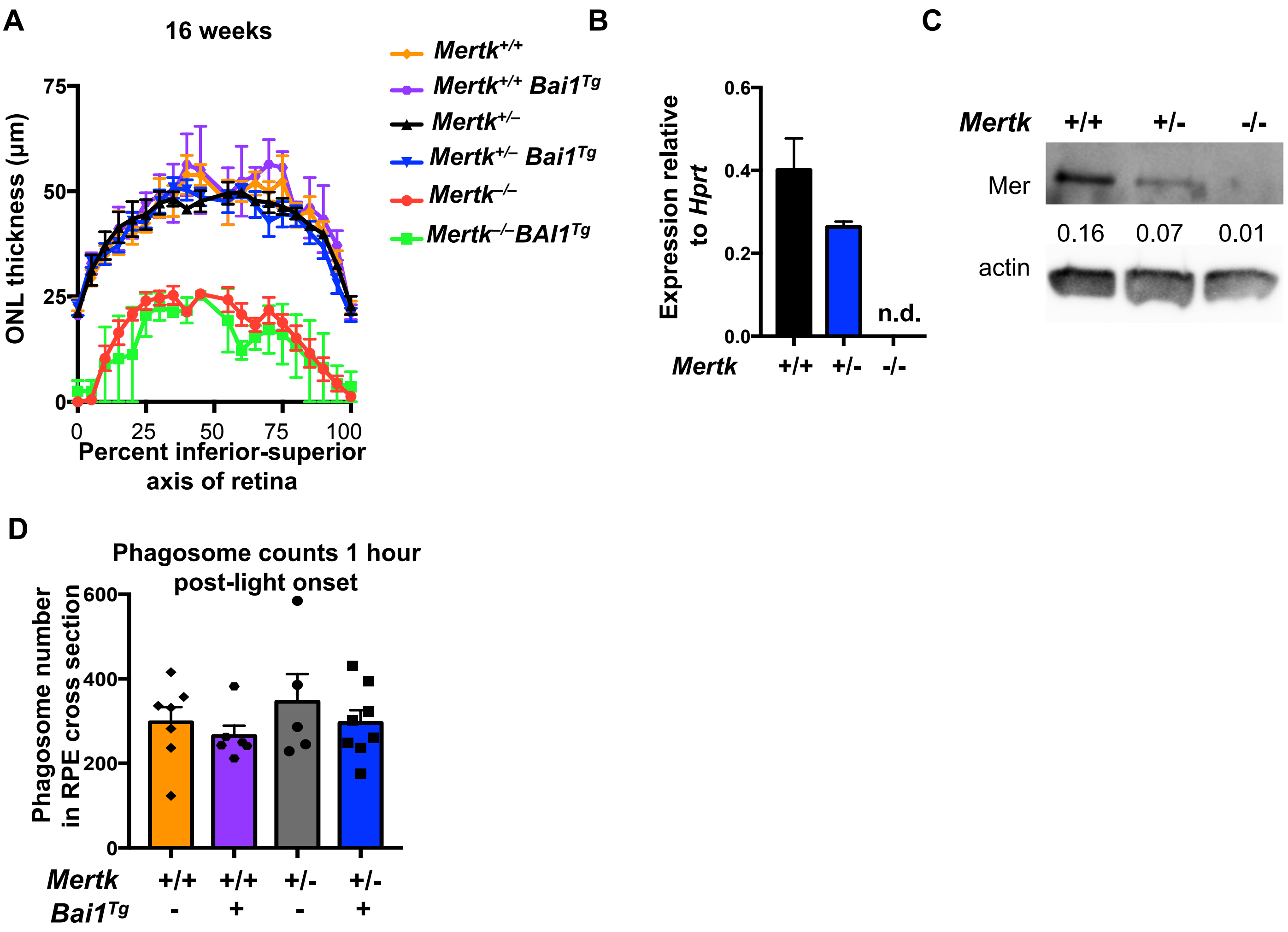

Figure S3: *Mertk*$^{+/+}$ and *Mertk*$^{+/-}$ mice display no phenotypic differences in retinal degeneration and phagocytosis

a) ONL thickness was measured in *Mertk*$^{+/+}$ (n=3), *Mertk*$^{+/+}$*Bai1*^{Tg} (n=2), *Mertk*$^{+/-}$ (n=4), *Mertk*$^{+/-}$*Bai1*^{Tg} (n=7), *Mertk*$^{-/-}$ (n=4) and *Mertk*$^{-/-}$*Bai1*^{Tg} (n=2) mice at 4 months of age.

b) *Mertk* expression in the RPE of *Mertk*$^{+/+}$ (n=2), *Mertk*$^{+/-}$ (n=2) and *Mertk*$^{-/-}$ (n=2) mice was evaluated by quantitative RT-PCR and

c) immunoblotting. Immunoblot shown is representative of 2 experiments.

d) Eyes from P17-P21 *Mertk*$^{+/+}$, *Mertk*$^{+/+}$*Bai1*^{Tg}, *Mertk*$^{+/-}$, *Mertk*$^{+/-}$*Bai1*^{Tg} mice were isolated at 1 h after light onset. Eyes were sectioned and stained for rhodopsin. Rhodopsin puncta in the RPE, referred to as phagosomes, were quantified by automated particle count in ImageJ. Symbols within the bars represent the average particle count from the left and right eyes of one mouse.

Figure S4: Bai1^{Tg} does not influence apical-basolateral localization of phagosomes

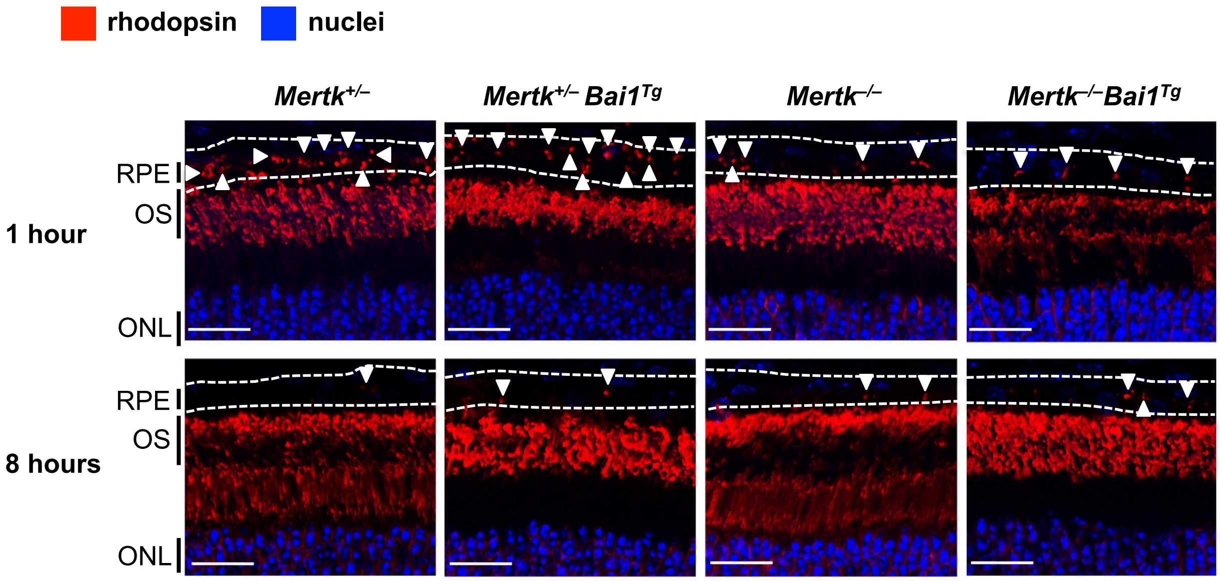

Figure S4: BAI1^{Tg} does not influence apical-basolateral localization of phagosomes
Eyecups isolated at 1 hour and 8 h after light onset were stained for rhodopsin and nuclei. Entire eye cups were imaged at 40x and phagosome localization was compared. Representative images from the central retina are shown. Dotted lines indicate RPE boundaries. White scale bars are 20μM. Rhodopsin punctae are indicated by white arrowheads.

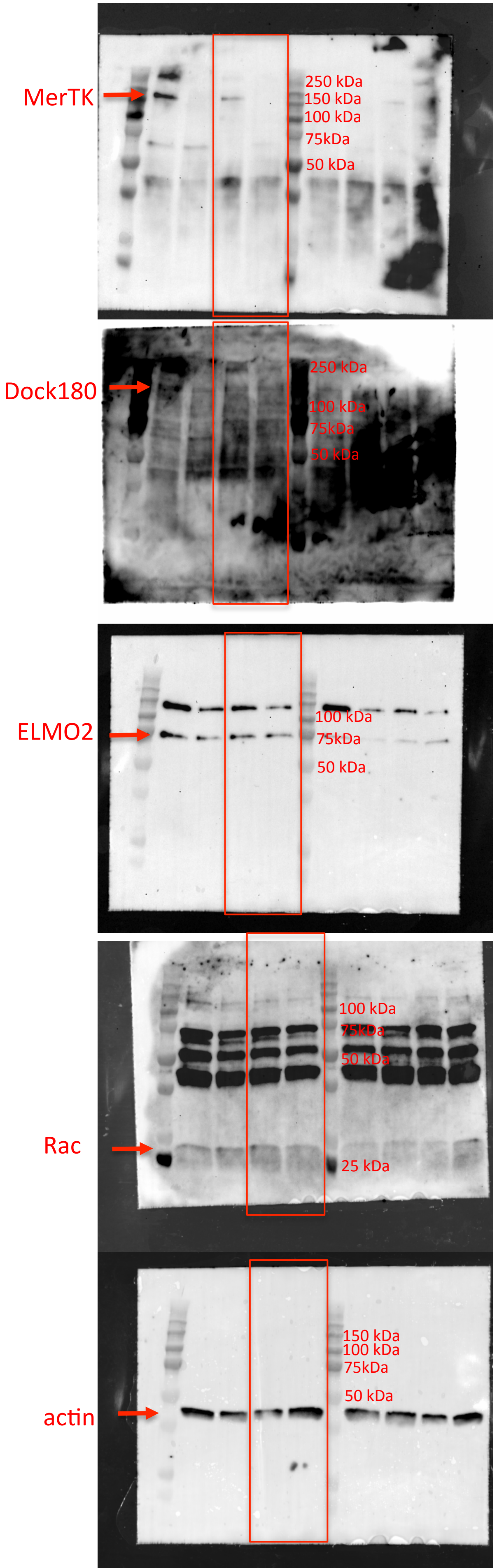
MerTK
250 kDa
150 kDa
100 kDa
75kDa
50 kDa
Dock180
250 kDa
100 kDa
75kDa
50 kDa
ELMO2
100 kDa
75kDa
50 kDa
Rac
100 kDa
75kDa
50 kDa
25 kDa
actin
150 kDa
100 kDa
75kDa
50 kDa

Figure 2B

Figure 2F

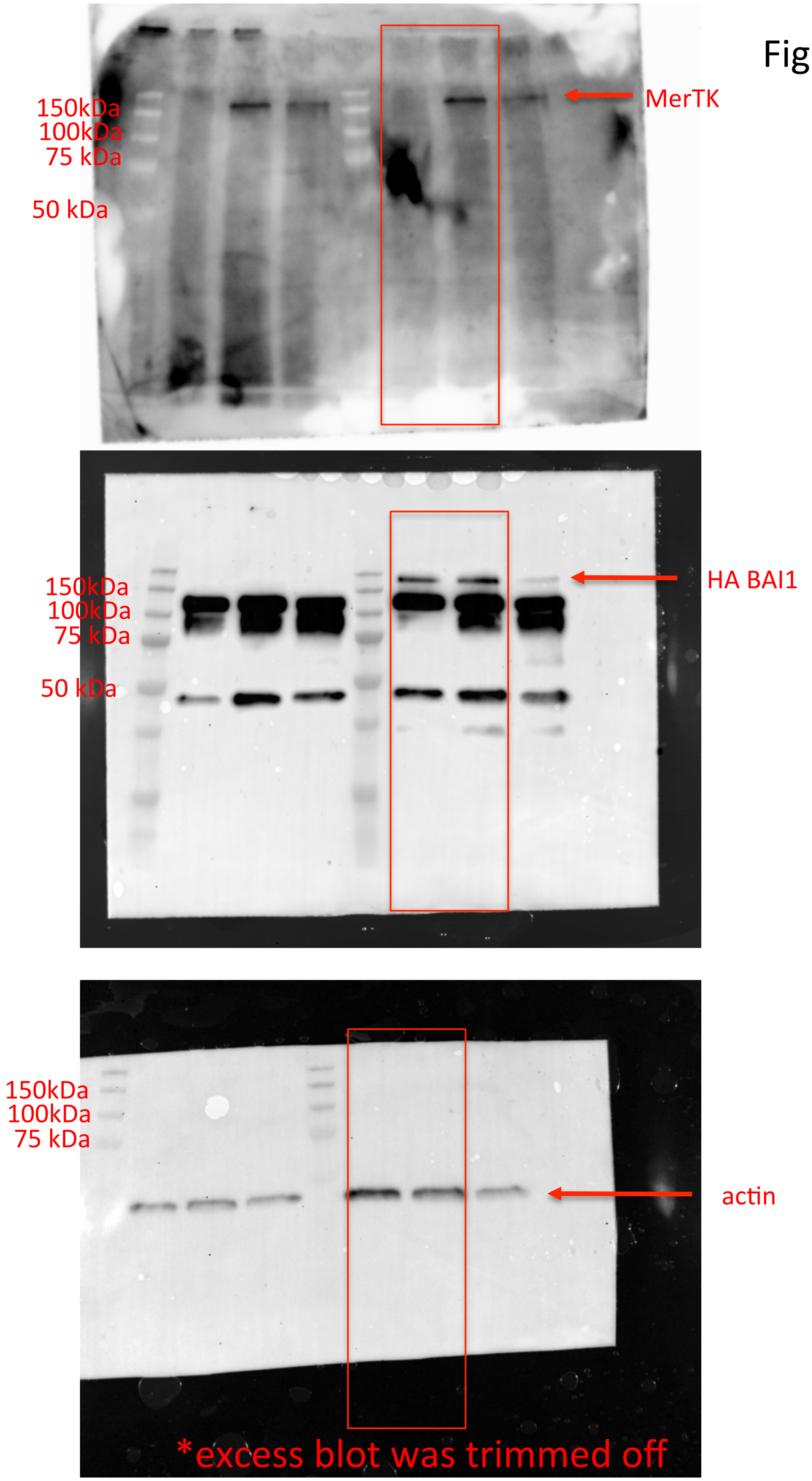
150kDa
100kDa
75 kDa
50 kDa
MerTK
150kDa
100kDa
75 kDa
50 kDa
HA BAI1
150kDa
100kDa
75 kDa
actin
*excess blot was trimmed off

Figure S3B

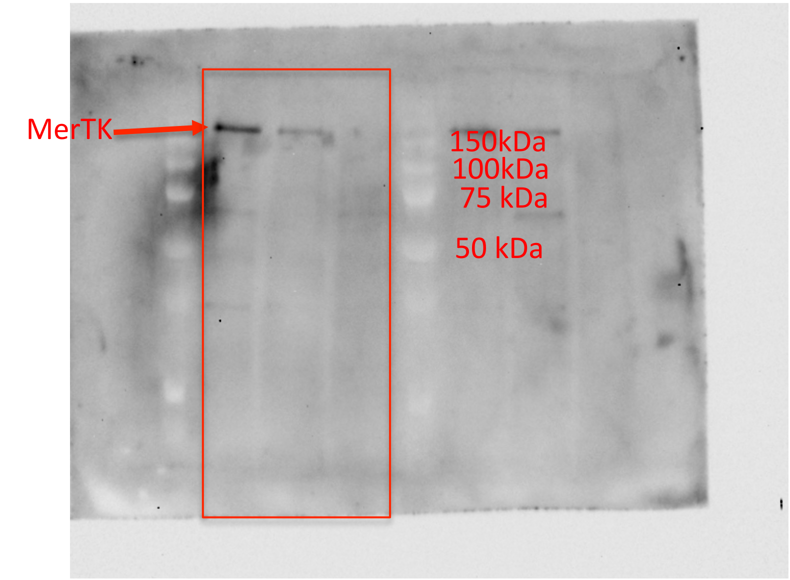
MerTK
150kDa
100kDa
75 kDa
50 kDa

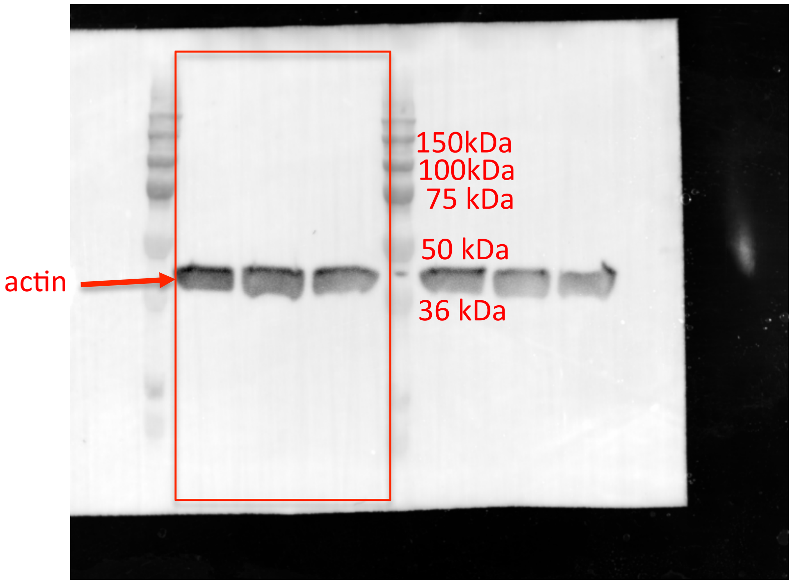
actin
150kDa
100kDa
75 kDa
50 kDa
36 kDa
